# New Appliances and Things Medical

**Published:** 1903-02-21

**Authors:** 


### NEW APPLIANCES AND THINGS MEDICAL.

[We shall be glad to receive at our Office, 28 & 29 Southampton Street, Strand, London, W.C., from the manufacturers, specimens of all new preparations and appliances which may be brought out from time to time.]

### BOYD'S ANTISEPTIC PERFUMES.

(THE MEDICAL AND GENERAL SPECIALITIES COMPANY, 300 CLAPHAM ROAD, S.W.)

THERE is no practical reason why ordinary domestic antiseptics for invalid and household use should not be made pleasantly odorous by the combination of suitable perfumes; indeed a large number of the essential oils which are employed in the preparation of perfumes have powerful germicide effects. The popular prejudice that to be efficacious an antiseptic must be malodiferous is founded on entirely wrong principles. Boyd's antiseptic perfumes, especially the lavender water and Eau de Cologne, are genuine examples of high-class perfumery, combined with antiseptic and deodorising qualities which for efficacy and efficiency are at least as reliable as many of the popular antiseptic preparations. Boyd's Eau de Cologne and lavender water may be strongly recommended.

### BOYD'S PINE-APPLE DIGESTIVE TONIC.

(MEDICAL AND GENERAL SPECIALITIES COMPANY., 300 CLAPHAM ROAD, S.W.)

THE attention of the medical profession has recently been drawn to the therapeutic properties of pine-apple, and the value of one of the active principles contained in the juice as an adjuvant to digestion. Boyd's Pine-apple Digestive Tonic, pine-hop tonic bitters, and phosphated pine-apple syrup are fine examples of artistic pharmacy, consisting as they do of therapeutic agents of considerable activity, combined with a delicious flavouring of pine-apple, together with valuable digestive properties of this fruit.

